# Mechanical, Electrical, and Biological Properties of Mechanochemically Processed Hydroxyapatite Ceramics

**DOI:** 10.3390/nano11092216

**Published:** 2021-08-28

**Authors:** Sujata Swain, Rakesh Bhaskar, Mukesh Kumar Gupta, Sonia Sharma, Sudip Dasgupta, Anuj Kumar, Pawan Kumar

**Affiliations:** 1 Department of Physics and Astronomy, NIT Rourkela, Rourkela 769008, India; sujataswain1994@gmail.com; 2 Department of Biotechnology and Medical Engineering, NIT Rourkela, Rourkela 769008, India; indiaxenobiotic@gmail.com (R.B.); guptam@nitrkl.ac.in (M.K.G.); 3 School of Chemical Engineering, Yeungnam University, Gyeongsan 38541, Korea; 4 Department of Chemistry, Govt. Autonomous College, Rourkela 769008, India; pvnsonia@gmail.com; 5 Department of Ceramic Engineering, NIT Rourkela, Rourkela 769008, India; dasguptas@nitrkl.ac.in

**Keywords:** hydroxyapatite, dielectric constant, cell proliferation, fracture toughness

## Abstract

The effect of the sintering temperature on densification and the resultant mechanical, electrical, and biological properties of mechanochemically processed hydroxyapatite (HAp) samples was investigated. HAp samples were sintered at 1200, 1250, and 1300 °C for 4 h, respectively. HAp samples sintered at 1250 °C showed better mechanical properties, which was attributed to their smaller grain size compared to HAp samples at higher sintering temperatures. The nearly identical value of the dielectric constant (ε_r_) and better cell proliferation was exhibited by HAp samples sintered at 1250 and 1300 °C, respectively. At ~210 °C, in all the samples sintered at different temperatures, a dielectric anomaly was obtained, which was attributed to the phase transition temperature of the HAp system. Dielectric properties near the phase transition temperature showed a dielectric relaxation-type of behavior, which was attributed to the re-orientational motion of OH^−^ ions in the HAp system. Higher cell proliferation and viability were exhibited by the HAp1300 samples, whereas comparatively equivalent cell growth and higher mechanical strength were observed in the HAp1250 samples.

## 1. Introduction

HAp, a calcium phosphate-based bioceramic, is an extensively used biomaterial for bone substitution [1]. This is because the HAp system has a similar chemical composition as that of human bones, excellent biocompatibility, and good osteoconductive properties, which make it one of the important implant materials for orthopedic-related treatments [2,3]. The HAp system also shows excellent stability in an aqueous medium above pH 4.3, which is within the range of the pH of human blood [4]. Several studies have shown that, for load-bearing applications, a dense form of HAp ceramic is required [5]. The well-defined particle morphology of HAp powder makes it quite effective in biomedical applications [4]. The microstructural, biological, and mechanical properties of the HAp system are mainly influenced by its particle size, crystallinity, morphology, stoichiometry, and composition. In this regard, synthesis precursors and processing techniques become important [6]. The HAp system starting powders in the nano range are obtained by different techniques like wet chemicals, sol-gel, precipitation, hydrolysis techniques, etc. The high-energy ball-milling (HEBM) method (also known as the mechanochemical method) is a novel technique used for obtaining nanoparticles of the starting precursors [7,8]. In terms of obtaining nano-size powders, the HEBM method has various advantages over other techniques, including being economical and highly scalable [9]. The HEBM technique involves mechanochemically activated solid-state reactions among the starting precursors. Nanoparticles, resulting from the mechanochemical method, have a narrow size distribution and low agglomeration [10]. The density of ceramics depends on sintering temperatures, keeping other factors like particle size, crystallinity, morphology, and starting powder constant [11]. Bone growth and fracture healing properties of the HAp system are related to its electrical properties [12,13,14]. Though cells are negatively charged, they are surrounded by a cloud of counter ions, which can migrate towards the cell surface under the application of the electrical field and can effectively set an induced dipole moment [15]. This results in polarization development in the HAp system. It has been reported that bone mass is linearly dependent on the HAp system’s dielectric properties [15]. This makes the study of dielectric properties of the HAp system imperative.

In the present work, the HEBM technique was used to obtain the nano-sized HAp system as starting powders. HAp samples were sintered at 1200, 1250, and 1300 °C for 4 h, respectively. Different properties like density; microstructure; and electrical, mechanical, and cellular activities on HAp samples, sintered at different temperatures, are characterized and discussed in detail.

## 2. Materials and Methods

Calcium carbonate (CaCO_3_, Merck) and ammonium dihydrogen phosphate ((NH_4_)H_2_PO_4_, Merck) were taken as the starting precursors for the synthesis of HAp powders. Initially, the stoichiometric proportion of starting precursors was taken in an aluminum vial for grinding with zirconia ball as a grinding medium. Mechanical activation was carried out through HEBM by maintaining 300 rpm for 5 h. After milling, raw powders were calcined at different temperatures (600 °C and 700 °C) in a conventional furnace with a heating rate of 5 °C/min. Seven hundred °C was optimized as the calcination temperature. Three wt% of PVA binder was added to the calcined HAp powders for better compaction, and green pellets were obtained by applying ~80 MPa pressure for 4 min in a hydraulic press. Sintering of the green pellets was carried out at 1200 °C, 1250 °C, and 1300 °C for 4 h, respectively, in a conventional furnace. Sintered HAp samples were electroded on both sides by applying silver paste and were subsequently heat-treated at 400 °C for good silver adhesion. In the present study, HAp samples, sintered at 1200 °C, 1250 °C, and 1300 °C temperatures are abbreviated as HAp1200, HAp1250, and HAp1300, respectively. HAp samples were characterized for density, phase composition, microstructure, hardness, diametral tensile strength, dielectric constant, and bioactivity. Phase confirmation of the calcined and sintered HAp samples was studied by using the X-ray diffraction method (Rigaku Ultima IV X-ray diffractometer) with Cu K_α_ radiation. Functional groups, present in the HAp system, were determined through FTIR spectroscopy by using an FTIR-Microscope-Shimadzu, IR prestige-21. Densities of the sintered samples were calculated by using Archimede’s principle by taking kerosene as the medium. The surface microstructures of the sintered samples were studied by using field emission scanning electron microscope (FESEM, NOVA Nano-SEM). The diametral tensile strength (σ) of the sintered HAp samples was measured by a universal testing machine by using the formula provided below [16]:

$$\sigma =\frac{2\times Fmax}{\pi \times D\times t}$$

where *F_max_* is the maximum breaking force, *D* is the diameter, and t is the thickness of the HAp samples.

The hardness (H_v_), fracture toughness (K_Ic_), and yield strength (Y) of the sintered HAp samples were measured by the Vickers micro-indentation method at a 1 kg load with a dwell time of 10 s. The K_Ic_ was calculated by using the formula provided below:

$$K_{\mathrm{Ic}}=0.203Ha^{1/2}{\left(\frac{c}{a}\right)}^{-3/2}$$

where *H* is the hardness, *c* is the radial crack length, measured from the center of the indent impression, and *a* is the half diagonal length of indentation [17]. The dielectric constant (ε_r_) and the dielectric loss (tanδ) variation of the sintered HAp samples as a function of frequency and temperature was carried out by using a computer-interfaced HIOKI 3352 system.

For in vitro cell–material interactions, an osteosarcoma cell line (MG-63, NCCS, Pune) was used. The MG-63 cells were routinely grown in an alpha minimum essential medium (α-MEM) supplemented with 10% (*v*:*v*) fetal bovine serum and antibiotics (Himedia, Mumbai, India) in T75 flasks (Corning, NY, USA) and were incubated at 37 °C for 1 to 7 days in a humidified atmosphere of 5% CO_2_ in air. For cell seeding, HAp samples were sterilized in 70% (*v*:*v*) ethanol, UV-exposed for 15–20 min, and washed several times with Dulbecco’s modified phosphate buffered saline (DPBS; pH 7.4). These HAp samples were then placed in 24-well polystyrene plates and treated with an antibiotic–antimycotic solution (Hi Media) for 15 min and left in complete media for 2–3 h. The MG-63 cells were seeded on the surface of HAp samples at a density of 5000/cm^2^ in each well. The plates were incubated in air at 37 °C in humidified atmosphere of 5% CO_2_. The culture media was changed every 2–3 days. Cell proliferation on HAp samples and tissue culture plates (positive control) were determined by using colorimetric MTT assay (3-(4,5-dimethylthiazol-2-yl)-2,5-diphenyltetrazolium bromide) for metabolic activity, using a commercial kit (Hi Media), as described elsewhere [18]. Briefly, after 1, 3, 5, and 7 days of cell seeding on HAp samples, cells were washed in the dark with DPBS and incubated with tetrazolium reagent for 4 h at 37 °C. The insoluble formazan was then dissolved in solubilization buffer, and absorbance of the purple color solution was measured at 595 nm using a spectrophotometer (Multiskan Go, Thermo Fisher Scientific, Vantaa, Finland). The OD595 values were corrected for blank (negative control; blank well containing the media only), normalized against the OD value of the day 1 TCP positive control, and plotted. Morphological study of MG-63 cells, grown on HAp samples, was performed for 1 to 7 days of cell culture by E-SEM, as described elsewhere [19]. Briefly, cell samples were rinsed twice with DPBS and fixed in 2.5% SEM-based glutaraldehyde for 3 h. HAp samples were further rinsed in SEM buffer and dehydrated with increasing concentrations of ethanol (50%, 70%, 90%, 95%, and 100%) for 3–4 min each. Finally, the cell samples were air-dried overnight and analyzed by E-SEM (FEI Quanta FEG 250). The viability of cells, seeded on HAp samples, was confirmed by evaluating the cytoplasmic esterase enzyme activity of cells by fluorescein diacetate-ethedium bromide (FDA-EtBr) staining and confocal laser scanning microscopy (Leica TCS SP8) after 5 and 7 days of culturing, as described elsewhere [20]. Briefly, the culture media was removed and washed twice with DPBS incubated with 2.5 mg/mL FDA and 10 µg/mL EtBr for 5 min in the dark. The stained cells were then washed with dye-free DPBS for at least two times to remove the excess traces of dyes, and they were observed under confocal microscopy (480–590 nm).

The hemocompatibility of the HAp samples was evaluated according to international standard of ISO 10993-4:2002, as described elsewhere [19]. Briefly, the blood was collected in ethylenediaminetetraacetic acid (EDTA) and diluted with normal saline (0.9% sodium chloride) in the ratio of 8:10 (*v*:*v*) as a working standard. Then, each HAp sample was taken in 0.5 mL of diluted blood, and the volume was increased to 10 mL with normal saline. The positive control was prepared by adding 0.5 mL of 0.1 M HCl to 9 mL of saline mixed with 0.5 mL of diluted blood, while the negative control was made by adding 0.5 mL of saline to 9 mL saline mixed with 0.5 mL diluted blood. The test samples, i.e., the HAp samples along with the positive and negative controls, were incubated at room temperature for 60 min, and after that they were centrifuged at 1000 rpm for 10 min and the absorbance (OD values) of supernatants were measured at 545 nm using spectrophotometer (Multiskan Go, Thermo Fisher Scientific, Vantaa, Finland). The percent hemolysis was determined by using the equation provided below:

Hemolysis (%) = (O.D_test_ − O.D_-ve control_)/(O.D_+ve contro_ − O.D_-ve control_) × 100%

## 3. Results and Discussion

### 3.1. XRD and FTIR Study

**Figure 1:**
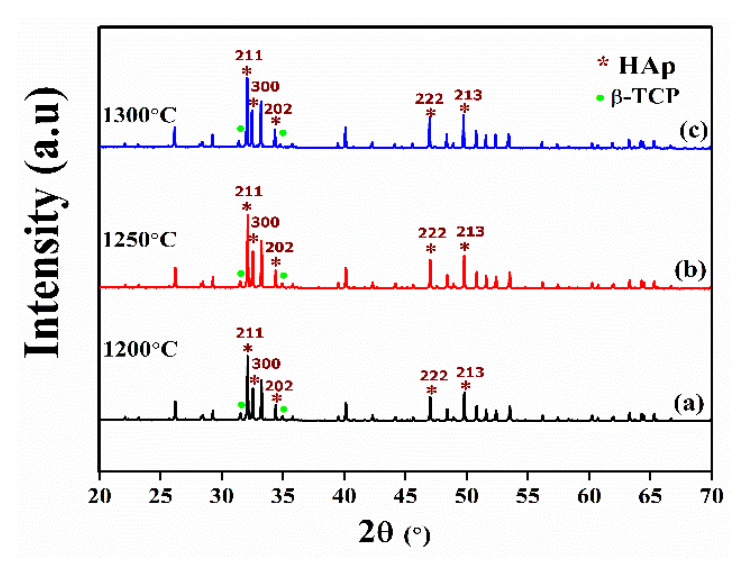
XRD pattern of HAp samples sintered at (**a**) 1200 °C, (**b**) 1250 °C, and (**c**) 1300 °C.

Figure 1 shows XRD patterns of HAp samples sintered at different temperatures. From XRD patterns, β-tricalcium phosphate (β-TCP) decomposition was observed in all the sintered HAp samples. Diffraction patterns of all the sintered samples mainly consisted of the hydroxyapatite phase and a very small amount (6–9%) of the β-TCP phase. Diffraction peaks were identified by using JCPDS card no. 76-0694 for the HAp phase and JCPDS card no. 09-0169 for the β-TCP phase. As reported earlier, formation of the β-TCP phase in the HAp system can be attributed to the loss of hydroxyl group at a higher sintering temperature [21].

The β-TCP phase can easily degrade and in turn supply Ca and P elements for the requirement of formation of ECM for newly generated bone tissue [22]. P. Sikder et al. also reported that a small amount of the β-TCP phase in the HAp system may favor the desired dissolution rate and bioactivity and enhance bone turnover by which the bonding between the HAp implant and natural bone is accelerated [23]. However, the β-TCP phase has lower mechanical properties, which limits its use in load-bearing applications. Therefore, an optimal amount of the β-TCP phase is desirable in the HAp system.

**Figure 2:**
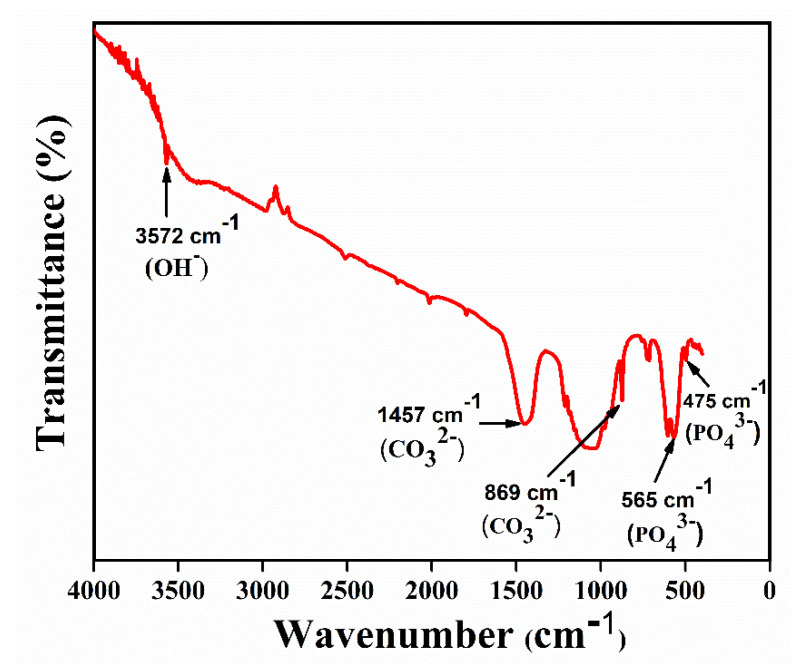
FTIR spectra of HAp powders.

Figure 2 shows FTIR spectroscopy of HAp powder calcined at 700 °C. The bands at ~3572 cm^−1^ confirm the presence of a hydroxyl group [24]. Likewise, other bands at ~475,565 cm^−1^ correspond to a phosphate group, and the bands at ~8,691,457 cm^−1^ correspond to a carboxyl group [25,26,27].

### 3.2. Density and Microstructure Study

**Figure 3:**
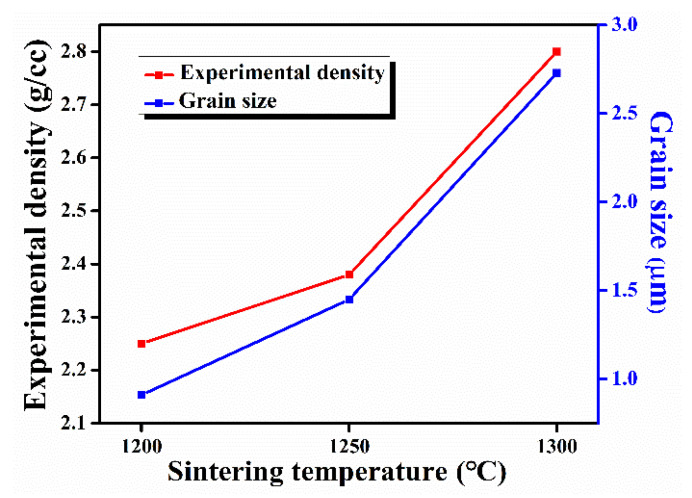
Density and grain size of sintered HAp samples sintered at different temperatures.

Figure 3 shows that with the increase in sintering temperature, the experimental density of HAp samples increased, and the highest density was found to be ~2.80 g/cc at a 1300 °C sintering temperature.

**Figure 4:**
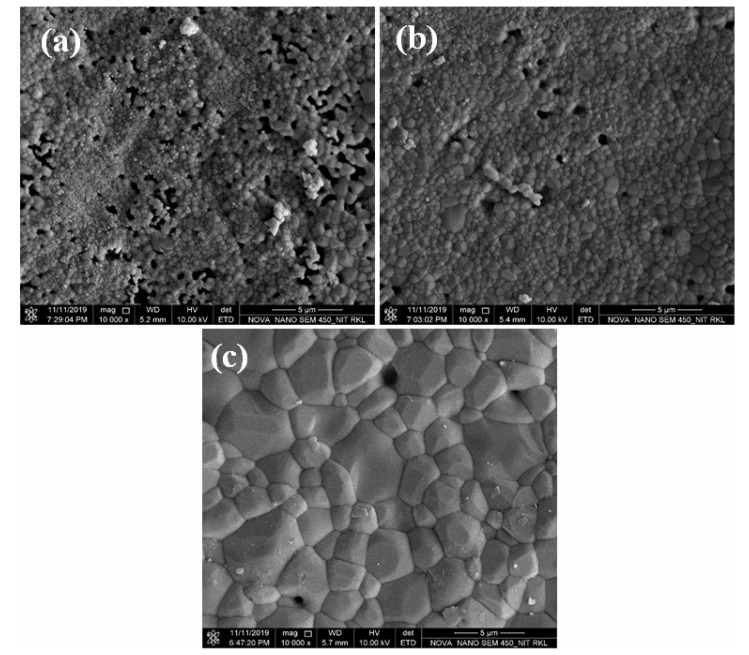
FESEM images of HAp samples sintered at (**a**) 1200 °C, (**b**) 1250 °C, and (**c**) 1300 °C.

Figure 4 shows the microstructure of HAp samples sintered at different temperatures. As shown in Figure 3, grain size increased with the increase in sintering temperature. The average grain size of the sintered HAp samples was found to be in between 0.9 and 2.73 μm.

The microstructure of the sintered samples was well correlated with the measured density. All the sintered HAp samples exhibited very less porosity, which decreased with the increase in the sintering temperature, as shown in the FESEM images. In the literature, it has been reported that porosity in the HAp system can accelerate the growth of bone tissues into the pores and can also provide an interlock for the fixation of an implant into the body [17]. Therefore, it will be interesting to observe the effect of porosity on the mechanical and bioactivity properties of sintered HAp samples.

### 3.3. Mechanical Properties Study

The value of diametral tensile strength (DTS), hardness, yield strength, and fracture toughness at different sintering temperature of the HAp system are provided in Table 1. The value of all these mechanical properties increases with the increase in sintering temperature up to 1250 °C, and after that it starts decreasing. It has been reported that enhanced mechanical properties were observed in an HAp system with a smaller grain size [17]. This is because the number of grain boundaries per unit volume increases with the decrease in grain size. Moreover, sintered compacts with a finer grain size offer more resistance to crack propagation and dislocation motion, which results in higher hardness and fracture toughness [24].

The hardness value may start decreasing above a certain critical grain size despite having a higher density of the sintered compacts [28]. Better mechanical strength, hardness, and fracture toughness was exhibited by HAp1250 samples compared to HAp1300 samples. The better mechanical properties of the HAp1250 samples can also be associated with the presence of relatively higher porosity than the HAp1300 samples. Sometimes, porosity plays a positive role in enhancing mechanical properties by absorbing the impact energy (accompanies crack splitting), which reduces crack propagation and thus delays rapid fracturing in the samples [29]. Additionally, mechanical properties as a function of sintering temperature depend on the combined effect of grain size and the density of the samples [30]. Porous materials usually have a lower mechanical strength compared to their dense counterparts [6]. However, an optimal combination of porosity, grain size, and density can result in better mechanical properties. The present study suggests that this optimal combination is present in HAp1250 samples. A fracture toughness of ~2.12 MPa.m^1/2^ was obtained in HAp 1250 samples, which was the highest among the reported literature on the HAp system [28].

### 3.4. Dielectric Study

**Figure 5:**
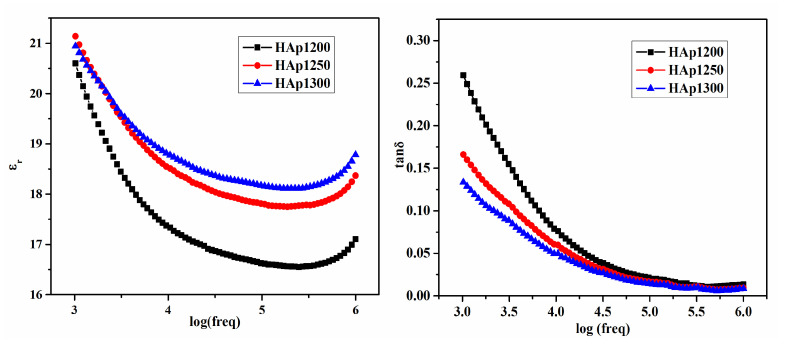
Variation in εr and tanδ with frequency at room temperature of sintered HAp samples.

Figure 5 shows the room temperature variation in ε_r_ and tanδ as a function of the frequency (1 kHz to 1 MHz) of the HAp samples, sintered at different temperatures. A decrease in ε_r_ with the increase in frequency was observed in all sintered samples, which can be attributed to decrease in net polarization. A high value of ε_r_ at a lower frequency can be associated with a higher space charge polarization, which was present in the samples.

It was observed that, at room temperature, with the increase in sintering temperature, ε_r_ increased and tanδ decreased. The simultaneous improvement of ε_r_ and the decrease in tanδ with the increase in sintering temperature can be related to the density and the grain size of the sintered HAp samples [31]. In the present study, it was observed that ε_r_ and tanδ of HAp1300 and HAp1250 samples were nearly equal. A high tanδ was found in the HAp1200 samples, which can be related to its higher porosity [32]. The highest value of ε_r_ ~21 at 1 kHz was exhibited by HAp1250 samples at room temperature.

**Figure 6:**
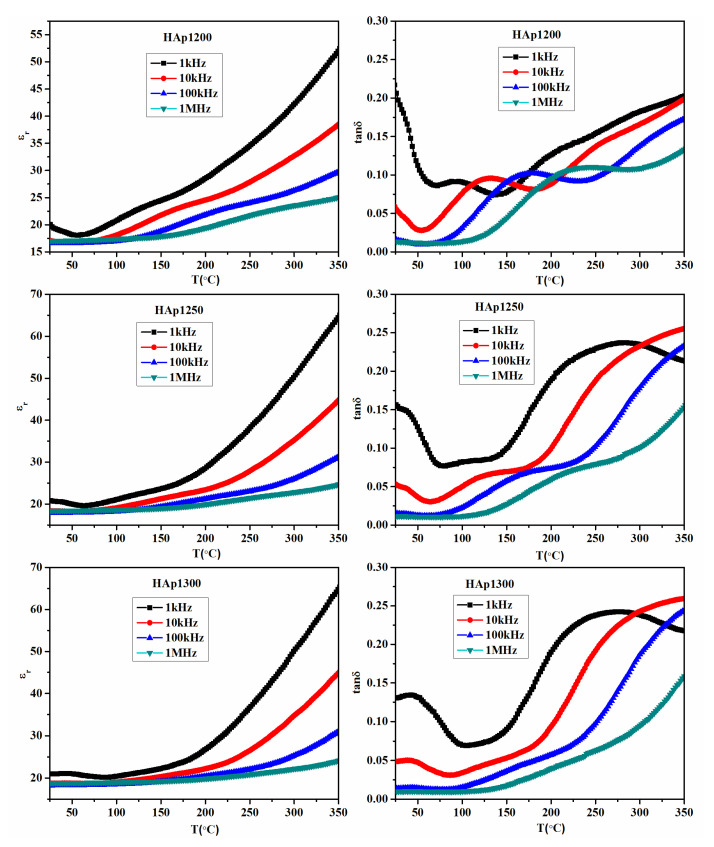
Variation in εr and tanδ (at different frequencies) with the temperature of sintered HAp samples.

Figure 6 shows the variation in ε_r_ and tanδ at different frequencies (1 kHz to 1 MHz) with the temperature of the sintered HAp samples. A prominent dielectric anomaly, which shifts towards higher temperatures with the increase in frequency, was observed in all the sintered samples. The dielectric anomaly in all the sintered HAp samples was observed at ~130 °C, ~175 °C, ~210 °C, and ~285 °C at 1 kHz, 10 kHz, 100 kHz, and 1 MHz frequencies, respectively. At ~210 °C, a dielectric anomaly was observed, which was also reported in the literature [33]. The dielectric anomaly of HAp samples at ~210 °C can be related to the re-orientational motion of OH^-^ ion dipoles.

As reported earlier, the mobility of OH^-^ ion dipoles change discontinuously at ~210 °C, which was the phase transition temperature of the HAp system, at which the monoclinic phase transforms to the hexagonal phase [34]. In the present study, above this phase transition temperature, a higher value of the dielectric constant was observed, which can be attributed to the better orientation of the OH^-^ ion dipoles in the hexagonal HAp phase than in the monoclinic HAp phase. Both ε_r_ and tanδ, near the phase transition temperature, showed a relaxation-type of behavior, which can be associated to the re-orientational motion of OH^-^ ions in the HAp system [35]. An increase in tanδ with the increase in temperature in all the sintered HAp samples can be attributed to the formation of free charge carriers/defects. At higher temperatures, the mobility of free charge carriers/defects increases, which gives rise to high tanδ in the HAp system [36].

### 3.5. Hemocompatibility, Cytocompatibility, and Cell Proliferative Effects

**Figure 7:**
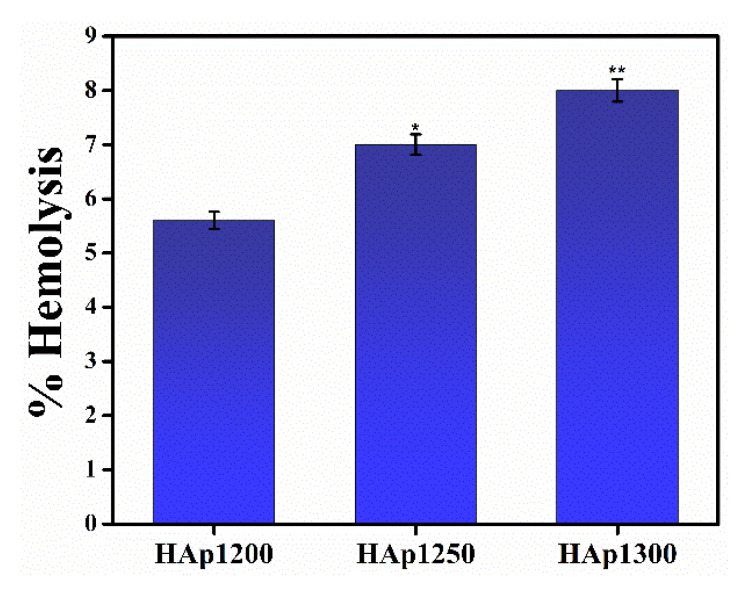
Hemocompatibility assay of HAp samples sintered at different temperatures. * and ** correspond to significant differences at *p* < 0.05 and *p* < 0.01, respectively.

HAp samples, to be used in the human body, must be hemocompatible. Therefore, it becomes important to carry out hemocompatibility assay study to evaluate if the synthesized HAp samples are compatible with the human body. It has been reported that the interaction of HAp samples with the blood may lead to damage of red blood cells, which may further cause anemia and blood coagulation [37]. Hemolysis% for all the sintered HAp samples was compared with the ASTM standard. As per the ASTM standard, samples were termed as highly hemocompatible (<5% hemolysis), hemocompatible (within 10% hemolysis), and non-hemocompatible (>20% hemolysis). It was observed that with the increase in sintering temperature, the hemolysis% of the HAp samples increased, as shown in Figure 7.

**Figure 8:**
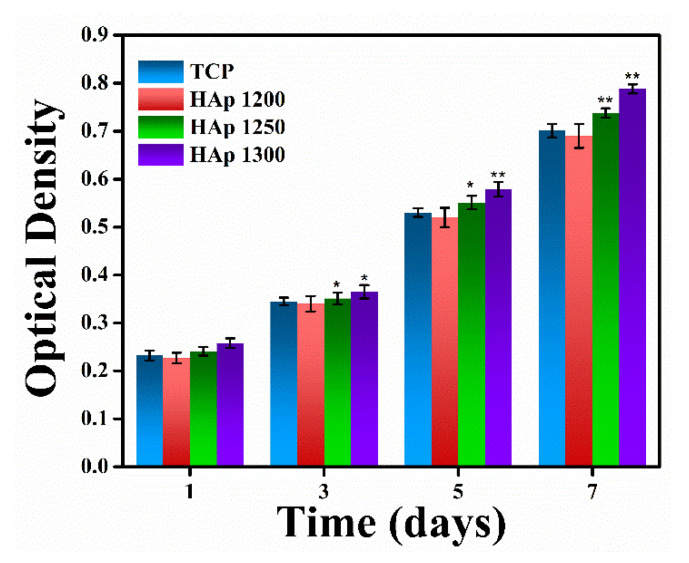
MTT assay of MG-63 cells cultured on HAp samples (sintered at different temperatures) for 7 days. * and ** correspond to significant differences at *p* < 0.05 and *p* < 0.01, respectively.

The hemolysis% was within the permissible level (below 10%) for hemocompatible materials. Thus, from the hemocompatibility study, it may be inferred that all the sintered HAp samples were hemocompatible in nature. The ability of HAp samples, sintered at different temperatures, to support cell proliferation was assessed by colorimetric MTT assay study, which estimates the mitochondrial activity of cells to catalyze the reduction in MTT to formazan by mitochondrial succinate dehydrogenase. An increase in formazan formation is indicative of an increase in the number of live cells. As expected, the number of cells increased with the culture duration in all samples, which thereby suggests that the HAp samples could support cell proliferation, as shown in Figure 8. Furthermore, there was a gradual increase in the proliferation rate with the increase in the sintering temperature of the HAp samples.

**Figure 9:**
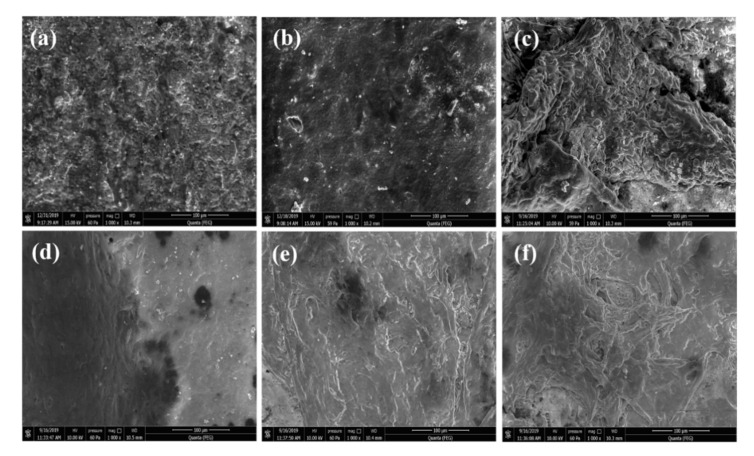
Morphology of HAp samples seeded with MG-63 cells. (**a**) HAp1200, (**b**) HAp1250, and (**c**) HAp1300 samples after 5 days; (**d**) HAp1200, (**e**) HAp1250, and (**f**) HAp1300 samples after 7 days.

Figure 9 shows a morphological study, analyzed by E-SEM, of MG 63 cells grown on sintered HAp samples for 5 and 7 days. Cell proliferation increased day by day, and a uniform formation of an apatite layer on the HAp samples’ surface was observed after 7 days. Multi-layer apatite formation was observed on the surface of HAp1300, whereas mono-layered apatite was observed on HAp1200. It has been reported that osseous tissues interact with the applied electrical field, i.e., electrical stimulation, and promote cell growth [38]. Additionally, the dielectric constant (ε_r_) of a material is directly proportional to the cellular biomass [15]. In the present study, it was observed that the value of ε_r_ for HAp1300 was higher compared to the other two fabricated samples (HAp1200 & HAp1250). It was speculated that the higher cell proliferation for HAp1300 was due to the multilayer formation of apatite on the surface.

**Figure 10:**
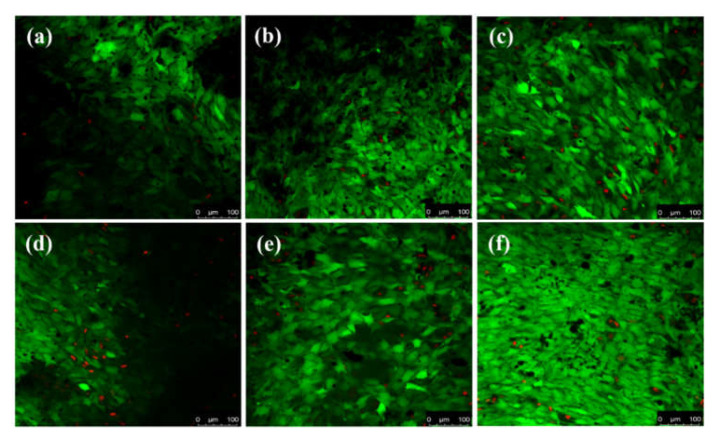
Viability of MG-63 cells grown on HAp samples. Viability was evaluated by cytoplasmic esterase enzyme activity and plasma membrane integrity, as measured by FDA-EtBr staining and confocal microscopy. The green color indicates live cells. (**a**) HAp1200, (**b**) HAp1250, and (**c**) HAp1300 samples after 5 days of culturing; (**d**) HAp1200, (**e**) HAp1250, and (**f**) HAp1300 samples after 7 days of culturing.

Figure 10 depicts confocal microscopy of HAp samples, sintered at different temperatures. Green fluorescence indicates live cells, whereas red fluorescence depicts dead cells. It was observed that with the increase in the culture period, the number of live cells increased, which supports the MTT and cell proliferation assay. After 5 and 7 days of culturing, the viability of cells on the HAP samples was observed, and it was found that HAp1300 samples showed the highest green fluorescence compared to other samples. Taken together with the MTT assay study, it may be concluded that HAP samples allow the cells to attach and proliferate without any cytotoxic effects.

## 4. Conclusions

HAp samples were successfully synthesized by an HEBM-assisted solid-state reaction route. The presence of a small amount of the β-TCP phase was observed in all the HAp samples, sintered at different temperatures. The highest room temperature ε_r_ ~21 at 1 kHz frequency was found in the HAp1250 samples. Dielectric properties above the phase transition temperature (210 °C) were associated with the motion of OH^−^ ion dipoles in different phases. Cell culture study indicated that the HAp1300 samples had higher cell proliferation and viability but with lower mechanical strength compared to the HAp1250 samples. Along with comparatively equivalent cell growth, better mechanical properties and a higher ε_r_ were observed in the HAp1250 samples, making it a suitable material for an alternate form of implant to be used for load-bearing applications.

## Figures and Tables

**Table 1:** Variation of mechanical properties with sintering temperature of HAp samples.

HAp Sintering Temperature (°C)	Diameter Tensile Strength (MPa)	Hardness (MPa)	Fracture Toughness (MPa.m^1/2^)	Yield Strength (MPa)
1200	10.21 ± 3.5	2075 ± 30.12	1.68 ± 0.08	691.66 ± 10.04
1250	14.42 ± 2.6	2654 ± 18.43	2.12 ± 0.05	884.66 ± 6.14
1300	8.82 ± 3.2	2356 ± 25.39	1.95 ± 0.03	785.33 ± 8.46

## Data Availability

Not applicable.
